# 

**Published:** 1903-05-30

**Authors:** 


### NOTICE TO CORRESPONDENTS.

All MSS., Letters, Books for Review, and other matters intended for the Editor should be addressed The Editor, "The Hospital," The Hospital Buildings, 28 & 29 Southampton Street, Strand, W.C.

The Editor cannot undertake to return rejected MS., even when accompanied by stamped directed envelope.

**Notice to Advertisers and Subscribers.**

All Advertisements, Orders for Copies of The Hospital, and Business Communications should be addressed to **THE MANAGER, (Not to the Editor)**, The Hospital, 28 & 29 Southampton Street, Strand, London, W.C.

The Cost of Subscription, post free, is as follows.—Per week, 3½d.; per quarter, 3s. 9d.; per half-year, 7s. 6d.; per annum, 15s. Foreign and Colonial:—Per annum, 19s. 6d., payable, in all cases, in advance.

**NOTICE.—Vol. XXXIII. of "The Hospital" (October 1902 to March 1903) in handsome cloth binding, will shortly be on sale at the office of this Journal, price 7s. 6d. Cases for binding the Numbers contained in this Volume 1s. 6d. Index to Vol. XXXIII., 2½d., post free.**

**The monthly part for June will be ready June 1st. Price 1s., by post 1s. 3d.**

### THE STUDENT OF MEDICINE.

### APPOINTMENTS.

**F. W. Beville**, L.R.C.P.Lond., M.R.C.S.Eng., appointed Certifying Factory Surgeon for the Denham District, Buckingham, and the Uxbridge District, Middlesex. **W. Brander**, M.B., Ch.B., appointed Resident-Assistant Medical Officer of the Ecclesall Bierlow Union Workhouse. **T. L. Butler**, M.R.C.S., L.R.C.P. Lond., appointed District Medical Officer of the Tavistock Union. **Henry Case**, L.R.C.P., L.R.C.S.Edin., appointed Public Vaccinator for the Withnell District of the Chorley Union. **J. R. Dickson**, M.B., C.M.Edin., appointed Assistant Medical Officer of Health and Bacteriologist of Trinidad. **G. B. Dixon**, M.R.C.S.Eng., L.R.C.P. Lond., appointed Assistant Medical Officer to the Walton Workhouse of the West Derby Union. **Arthur E. Dodson**, M.R.C.S., etc., appointed Medical Officer to the Intermediate Schools of the Wandsworth and Clapham Union. **David Fleck**, M.B., B.Ch., B.A.O. (R.U.I.), M.P.C., appointed Resident Superintendent of the Royal Victoria Homes, Brentry. **O. Johnson**, L.R.C.P., L.R.C.S. Edin., L.F.P.S.Glasg., appointed District Medical Officer of the Lincoln Union. **Duncan D. Mackintosh**, M.B., C.M.Aberd., appointed Medical Officer, Parish of Lumphanan, also Certifying Factory Surgeon for the Aboyne District, Aberdeenshire. **R. A. M. Macleod**, M.B., C.M.Edin., appointed Assistant Medical Officer to the West Derby Union Infirmary. **H. Prytherch**, L.R.C.P. Edin., M.R.C.S.Eng., appointed Certifying Factory Surgeon for the Beaumaris District, Anglesey.

### The Untrained

*versus*

### The Trained Nurse.

The untrained nurse is no longer countenanced, we are rapidly leaving the days of Mrs. Gamp far behind us. It is necessary therefore that the nurse aspirant should before entering upon this important calling obtain the fullest particulars as to **how and where to train** in order to become an efficient nurse. In

### THE NURSING PROFESSION,

By Sir HENRY BURDETT, K.C.B.,

Price **2/4** post free,

The would-be nurse will find **all** the information she needs in deciding upon the Institution at which to go through her training and to find employment afterwards. The book is up to date in all respects, and is in fact indispensable to anyone wishing to become a nurse and to be properly trained.

---

### Aids to Training.

### NURSING: ITS THEORY AND PRACTICE,

By Percy G. Lewis, M.D., price **3/6** post free,
Is a complete Text-book of Medical, Surgical and Monthly Nursing.

### ELEMENTARY ANATOMY AND SURGERY FOR NURSES,

By William McAdam Eccles, M.S. Lond., M.B., price **2/6** post free,
Is invaluable to Nurses who are beginning the study of Anatomy.

### ELEMENTARY PHYSIOLOGY FOR NURSES,

By C. F. Marshall, M.D., B.Sc., F.R.C.S., price **2/-** post free,
Contains all that Nurses require to know of this subject.

### THE NURSE'S DICTIONARY OF MEDICAL TERMS,

By Honnor Morten, price **2/-** post free,
Should always be carried in the apron pocket for ready reference.

---

*These books are obtainable of all Booksellers and are published by*
THE SCIENTIFIC PRESS, LIMITED, 28 & 29 Southampton Street, Strand, London,
*who will send their latest Catalogue of Nursing Text-books post free to any Nurse on application.*

